# Multi-Slice CT Features Predict Pathological Risk Classification in Gastric Stromal Tumors Larger Than 2 cm: A Retrospective Study

**DOI:** 10.3390/diagnostics13203192

**Published:** 2023-10-12

**Authors:** Sikai Wang, Ping Dai, Guangyan Si, Mengsu Zeng, Mingliang Wang

**Affiliations:** 1 Department of Radiology, The Affiliated Traditional Chinese Medicine Hospital of Southwest Medical University, No. 182 Chunhui Road, Longmatan District, Luzhou 646000, China; qiuzhi2010@163.com (S.W.); daipbb0830@163.com (P.D.); 2 Department of Radiology, Zhongshan Hospital, Fudan University, No. 180 Fenglin Road, Xuhui District, Shanghai 200032, China; zeng.mengsu@zs-hospital.sh.cn

**Keywords:** gastric stromal tumors, X-ray computed, risk classification, Armed Forces Institute of Pathology, nomogram model

## Abstract

Background: The Armed Forces Institute of Pathology (AFIP) had higher accuracy and reliability in prognostic assessment and treatment strategies for patients with gastric stromal tumors (GSTs). The AFIP classification is frequently used in clinical applications. But the risk classification is only available for patients who are previously untreated and received complete resection. We aimed to investigate the feasibility of multi-slice MSCT features of GSTs in predicting AFIP risk classification preoperatively. Methods: The clinical data and MSCT features of 424 patients with solitary GSTs were retrospectively reviewed. According to pathological AFIP risk criteria, 424 GSTs were divided into a low-risk group (*n* = 282), a moderate-risk group (*n* = 72), and a high-risk group (*n* = 70). The clinical data and MSCT features of GSTs were compared among the three groups. Those variables (*p* < 0.05) in the univariate analysis were included in the multivariate analysis. The nomogram was created using the rms package. Results: We found significant differences in the tumor location, morphology, necrosis, ulceration, growth pattern, feeding artery, vascular-like enhancement, fat-positive signs around GSTs, CT value in the venous phase, CT value increment in the venous phase, longest diameter, and maximum short diameter (all *p* < 0.05). Two nomogram models were successfully constructed to predict the risk of GSTs. Low- vs. high-risk group: the independent risk factors of high-risk GSTs included the location, ulceration, and longest diameter. The area under the receiver operating characteristic curve (AUC) of the prediction model was 0.911 (95% CI: 0.872–0.951), and the sensitivity and specificity were 80.0% and 89.0%, respectively. Moderate- vs. high-risk group: the morphology, necrosis, and feeding artery were independent risk factors of a high risk of GSTs, with an AUC value of 0.826 (95% CI: 0.759–0.893), and the sensitivity and specificity were 85.7% and 70.8%, respectively. Conclusions: The MSCT features of GSTs and the nomogram model have great practical value in predicting pathological AFIP risk classification between high-risk and non-high-risk groups before surgery.

## 1. Introduction

Gastrointestinal stromal tumors (GISTs) are very rare but are the most common mesenchymal tumors of the digestive tract. GISTs occur most frequently in the stomach (50–60%) and small intestine (20–30%) [1]. Gastric stromal tumors (GSTs) differ in the range of biological behavior from benign to extremely malignant. With the development of targeted drug therapy, accurate risk stratification for GSTs has become increasingly important. Presently, different risk classification standards are used for GSTs. The 2008 modified version of the National Institutes of Health (NIH) classification and the Armed Forces Institute of Pathology (AFIP) classification are frequently used in clinical applications [2,3]. Previous studies [3,4,5,6] showed that AFIP criteria had higher accuracy and reliability in prognostic assessment and treatment strategies for patients with GSTs.

AFIP risk stratification was typically based on the tumor location, size, and mitotic count [7]. However, due to tumor stroma changes induced by treatment with tyrosine kinase inhibitor (TKI) before surgery, the mitotic count and tumor size cannot be accurately evaluated using post-operative specimens. Preoperative puncture biopsy may also not provide accurate measurements of mitotic count when samples are few and for more heterogeneous tumors. Moreover, puncture biopsy may cause the tumor to rupture, bleed, or seed, spreading cancer cells along the needle path. Therefore, the risk classification is only available for patients who were previously untreated and received complete resection.

MSCT has emerged as a clinically preferred imaging modality for its ability to provide a differential diagnosis, an evaluation of metastasis and therapy, and a prediction of rupture and follow-up after surgery [1,8,9]. Previous comparative studies [8,10,11] reported different risk classification and prognosis evaluations of GISTs using CT or other inspection methods. However, most of these studies included all GISTs and had fewer samples. Liu et al. [12,13] reported that CT and clinical features differed between GSTs and non-GSTs. In addition, GSTs account for more than half of GISTs. Accurate preoperative risk assessment of GSTs using MSCT has important clinical significance in guiding treatment and predicting prognosis. Therefore, this study aims to investigate the feasibility of MSCT features in predicting pathological AFIP risk classification of GSTs before treatment.

## 2. Methods

### 2.1. Study Population

The clinical data and MSCT features of 476 patients with GSTs confirmed by surgical and post-operative pathological examination at Zhongshan Hospital, Fudan University and the Affiliated Traditional Chinese Medicine Hospital of Southwest Medical University were retrospectively reviewed from November 2014 to November 2021.

The inclusion criteria: (1) GSTs were resected completely and were confirmed by the post-operative pathological examination; (2) patients underwent abdominal triple-phase (non-contrast CT, arterial, and venous phases) CT scan before surgery; and (3) longest diameter of tumor specimen > 2 cm.

**Figure 1:**
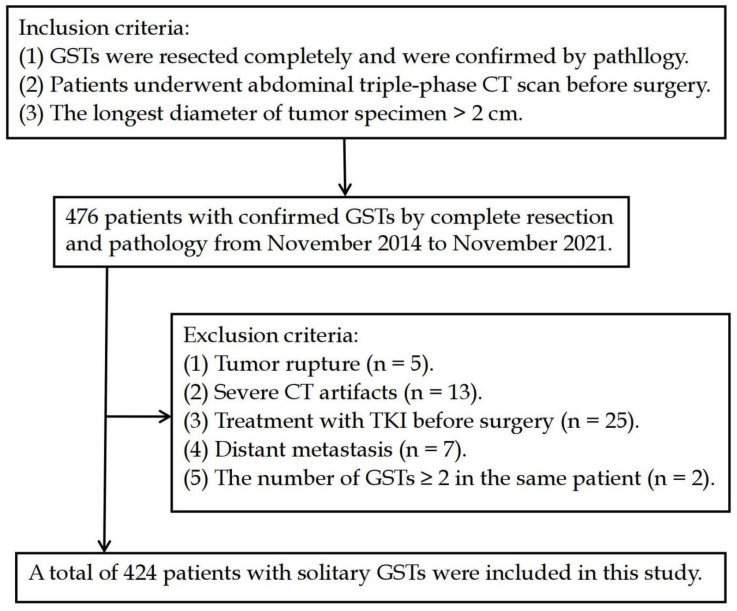
Flow diagram of the study population.

The exclusion criteria: (1) tumor rupture (preoperative or intraoperative tumor rupture; tumor specimens were several pieces or incomplete); (2) severe CT artifacts; (3) treatment with TKI before surgery; (4) distant metastasis (including lymph node metastasis, visceral metastases, peritoneal metastases) was confirmed by biopsy; and (5) the number of GSTs ≥ 2 in the same patient (Figure 1).

After screening, a total of 424 patients with solitary GSTs were included in this study. According to the AFIP criteria [7] (Table 1), 424 GSTs were categorized as a low-risk group (including very low and low risk), a moderate-risk group, and a high-risk group.

### 2.2. CT Technique

All the patients drank 500–800 mL of water 15 min before the CT examination to expand the stomach and were trained in breathing. All the patients underwent a triple-phase (non-contrast, arterial, and venous phases) CT scan using one of the following MSCT scanners: 64-slice spiral CT (Siemens Medical Solutions, Forchheim, Germany), second Dual Source CT (Siemens Medical Solutions, Forchheim, Germany), or UIH 40 CT (United Imaging Healthcare, Shanghai, China) scanner. The scanning parameters were as follows: 120-kV tube voltage, adaptive tube current, and axial images of 5 mm slice thickness. An 80–100 mL dose of non-ionic contrast agent (iopromide, 370 mgI/mL iodine, Bayer Schering Company, Guangzhou, China; or iohexol, 300 mgI/mL iodine, GE Healthcare, Shanghai, China) was injected into the cubital vein at a rate of 2.5–3 mL/s using a dual-barrel power injector. The scan of the arterial and venous phases was initiated at about 25 s and 80 s after starting the contrast injection, respectively.

### 2.3. Imaging Interpretation

Two radiologists with more than 10 years of clinical experience in abdominal CT diagnosis reviewed the CT images using a single-blinded comparison to reach an agreement through consultation, without prior knowledge of the pathological results. A consensus was reached between a third senior abdominal radiologist and the two radiologists when there was any disagreement between the two radiologists.

The following quantitative parameters were used in this study: (1) longest diameter on axial image, maximal short diameter (perpendicular to longest diameter on the same axial image); (2) CT value in non-contrast CT, arterial phase, and venous phase. More than 20 mm^2^, circular regions of interest (ROI) of triple-phase CT value were drawn on the same solid parts of lesions while avoiding necrotic cystic areas, calcification, and vascular areas; (3) CT value increment in arterial phase (ΔCT_arterial_) was calculated by subtracting the CT value in non-contrast CT from the CT value in the arterial phase; (4) CT value increment in venous phase (ΔCT_venous_) was calculated by subtracting the CT value in the non-contrast CT scan from the CT value in the venous phase.

The qualitative parameters were as follows: (1) morphology (regular was defined as smooth-walled, round, or oval; irregular otherwise); (2) growth pattern (endophytic vs. exophytic vs. mixed); (3) calcification (defined as extremely high-density imaging in the non-contrast CT); (4) ulceration (defined as superficial defects of tumor); (5) necrosis (defined as unenhanced regions in arterial and venous phase); (6) location (gastric fundus vs. gastric cardia vs. gastric body vs. gastric antrum); (7) feeding artery (larger arteries enter the tumor in the arterial phase); (8) vascular-like enhancement (striated vascular shadow was seen in the arterial phase or venous phase inside the tumor); (9) fat-positive signs around the lesion (increased fat density).

### 2.4. Statistical Analyses

SPSS statistical software (version: 26.0) and R software (version: 4.0.3) were used to process and analyze all the data. All the continuous variables that did not always follow a normal distribution were presented as the median (first quartile, third quartile). The quantitative data were statistically analyzed using the Kruskal–Wallis H test, and the least significant difference (LSD) test was used for pairwise comparisons. The categorical variables were expressed as frequencies (percentages). The categorical variables were statistically analyzed using the chi-square test and Bonferroni method for pairwise comparisons. Those variables (*p* < 0.05) in the univariate analysis were included in the multivariate analysis. Multivariate analysis was performed with stepwise logistic regression based on the Akaike information criterion. The nomogram was created using the rms package. The calibration curve (the Brier score) and receiver operating characteristic (ROC) curve were used to evaluate the predictive performance of the nomogram model. A smaller value of the Brier score (<0.2) suggests a better model. The bootstrap resampling method (1000 samples) was used for internal validation and the stability of the model. The random seed was set to 123,456. *p* < 0.05 was considered statistically significant.

## 3. Results

### 3.1. Clinical Information (Table 2)

A total of 424 patients (223 men and 201 women) with a median age of 61.0 (range, 14–85) years were included in the study. In total, 424 GSTs were divided into a low-risk group (*n* = 282), a moderate-risk group (*n* = 72), and a high-risk group (*n* = 70). We found no significant differences in age, gender, and gastrointestinal bleeding among the three groups (*p* > 0.05).

**Table 2:** Comparison of clinical information.

Groups	Gender		Age (Years Old)	Gastrointestinal Bleeding	
	Male (*n* = 223)	Female (*n* = 201)		Yes (*n* = 96)	No (*n* = 328)
Low-risk group (*n* = 282)	146(51.8)	138 (48.2)	61.00 (54.00, 69.00)	55 (19.5)	227 (80.5)
Moderate-risk group (*n* = 72)	32 (44.4)	40 (55.6)	60.50 (52.50, 68.00)	19 (26.4)	53 (73.6)
High-risk group (*n* = 70)	45 (64.3)	25 (35.7)	60.00 (52.00, 67.00)	22 (31.4)	48 (68.6)
Statistical Value	5.832 ^#^		1.181 *	5.248 ^#^	
*p* value	0.054		0.554	0.072	

Note: * represents H value, ^#^ represents χ^2^ value.

### 3.2. CT Features (Table 3, Figure 2)

There were significant differences in the longest diameter, maximum short diameter, tumor location, morphology, necrosis, ulceration, growth pattern, feeding artery, vascular-like enhancement, fat-positive signs around the lesion, the venous phase CT value, and the ΔCT_venous_ among the three groups (*p* < 0.05). However, we found no significant differences in the CT value in the non-contrast and arterial phase, ΔCT_arterial_, and calcification (*p* > 0.05).

**Figure 2:**
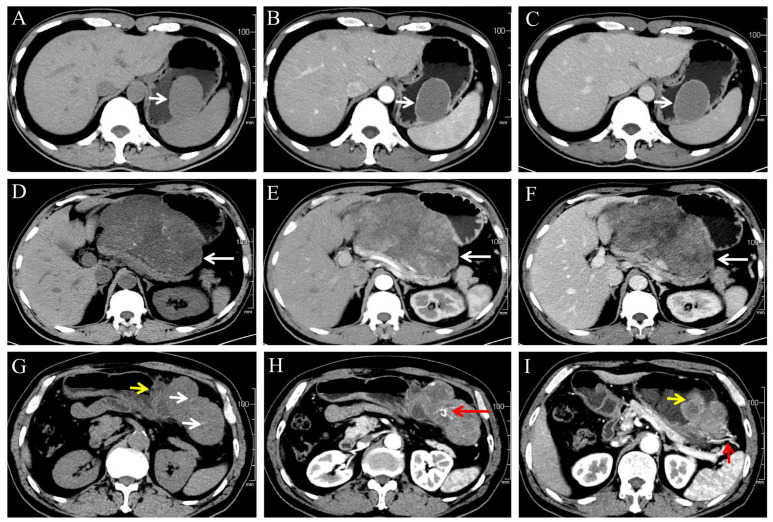
(**A**–**C**) A 46-year-old male patient with very low-risk GSTs in gastric body (white arrow). The non-contrast (**A**), arterial phase (**B**), and venous phase (**C**) CT images showed endophytic growth tumor with regular morphology, homogeneous enhancement and no necrosis. (**D**–**F**) A 59-year-old male patient with moderate-risk GSTs in gastric body (white arrow). The non-contrast (**D**), arterial phase (**E**), and venous phase (**F**) CT images showed exophytic growth lesion with irregular morphology, thin dotted calcification (**D**), heterogeneous enhancement, and necrosis. (**G**–**I**) A 57-year-old male patient with high-risk GSTs in gastric fundus. The non-contrast (**G**), arterial phase (**H**,**I**) CT images showed a mixed growth tumor with irregular morphology, thin dotted calcification ((**G**), white arrow), heterogeneous enhancement and necrosis area, feeding artery ((**I**), short red arrow), vascular-like enhancement ((**H**), long red arrow), and ulceration ((**G**,**I**), short yellow arrow).

**Table 3:** Comparison of MSCT features of GSTs.

Parameters	Low-Risk Group (*n* = 282)	Moderate-Risk Group (*n* = 72)	High-Risk Group (*n* = 70)	Statistical Value	*p* Value
Morphology					
Regular (*n* = 239)	178 (63.1) ^a^	46 (63.9) ^a^	15 (21.4) ^b^	41.629 ^#^	<0.001
Irregular (*n* = 185)	104 (36.9)	26 (36.1)	55 (78.6)		
Calcification					
No (*n* = 346)	232 (82.3)	61 (84.7)	53 (75.7)	44.338 ^#^	0.338
Yes (*n* = 78)	50 (17.7)	11 (15.3)	17 (24.3)		
Ulceration					
No (*n* = 299)	228 (80.9) ^a^	42 (58.3) ^b^	29 (41.4) ^c^	48.116 ^#^	<0.001
Yes (*n* = 125)	54 (19.1)	30 (41.7)	41 (58.6)		
Feeding artery					
No (*n* = 294)	216 (76.6) ^a^	55 (76.4) ^a^	23 (32.9) ^b^	52.490 ^#^	<0.001
Yes (*n* = 130)	66 (23.4)	17 (23.6)	47 (67.1)		
Vascular-like enhancement					
No (*n* = 321)	233 (82.6) ^a^	57 (79.2) ^a^	31 (44.3) ^b^	45.383 ^#^	<0.001
Yes (*n* = 103)	49 (17.4)	15 (20.8)	39 (55.7)		
Fat-positive sign around lesion					
No (*n* = 394)	275 (97.5) ^a^	69 (95.8) ^a^	50 (71.4) ^b^	59.171 ^#^	<0.001
Yes (*n* = 30)	7 (2.5)	3 (4.2)	20 (28.6)		
Necrosis					
No (*n* = 183)	142 (50.4) ^a^	36 (50.0) ^a^	5 (7.1) ^b^	44.338 ^#^	<0.001
Yes (*n* = 241)	140 (49.6)	36 (50.0)	65 (92.9)		
Location					
Gastric fundus (*n* = 157)	90 (31.9) ^a^	38 (52.8) ^b^	29 (41.4) ^ab^	29.563 ^#^	<0.001
Gastric cardi (*n* = 10)	4 (1.4) ^a^	1 (1.4) ^ab^	5 (7.1) ^b^		
Gastric body (*n* = 215)	149 (52.8) ^a^	31 (43.1) ^a^	35 (50.0) ^a^		
Gastric antrum (*n* = 42)	39 (13.8) ^a^	2 (2.8) ^b^	1 (1.4) ^b^		
Growth pattern					
Endophytic (*n* = 133)	81 (28.7) ^a^	25 (34.7) ^a^	7 (10.0) ^b^	19.755 ^#^	0.001
Exophytic (*n* = 179)	124 (44.0) ^a^	27 (37.5) ^a^	28 (40.0) ^a^		
Mixed (*n* = 132)	77 (27.3) ^a^	20 (27.8) ^a^	35 (50.0) ^b^		
Maximal short diameter (cm)	2.87 (2.30, 3.84) ^a^	3.07 (2.36, 3.99) ^a^	6.06 (4.34, 8.29) ^b^	98.088 *	<0.001
CT value in non-contrast (HU)	32.45 (29.00, 36.00)	32.85 (29.45, 34.60)	33.55 (30.30, 36.00)	1.740 *	0.419
CT value in arterial phase (HU)	52.75 (46.00, 60.70)	48.95 (46.13, 57.15)	49.95 (44.40, 58.00)	3.504 *	0.173
CT value in venous phase (HU)	64.10 (57.40, 74.20) ^a^	61.05 (54.80, 67.75) ^b^	59.50 (54.05, 69.40) ^b^	9.208 *	0.010
ΔCT_arterial_	19.50 (13.39, 26.70)	17.83 (12.65, 24.38)	16.50 (11.75, 23.93)	4.193 *	0.123
ΔCT_venous_	31.00 (24.60, 41.49) ^a^	27.55 (21.50, 37.60) ^b^	26.25 (21.12, 36.15) ^b^	8.557 *	0.014

Note: * represents H value, ^#^ represents χ^2^ value, Different letters (^a, b, c^) indicate statistical differences.

### 3.3. Construction of Binary Logistic Regression and Nomogram Model

**Figure 3:**
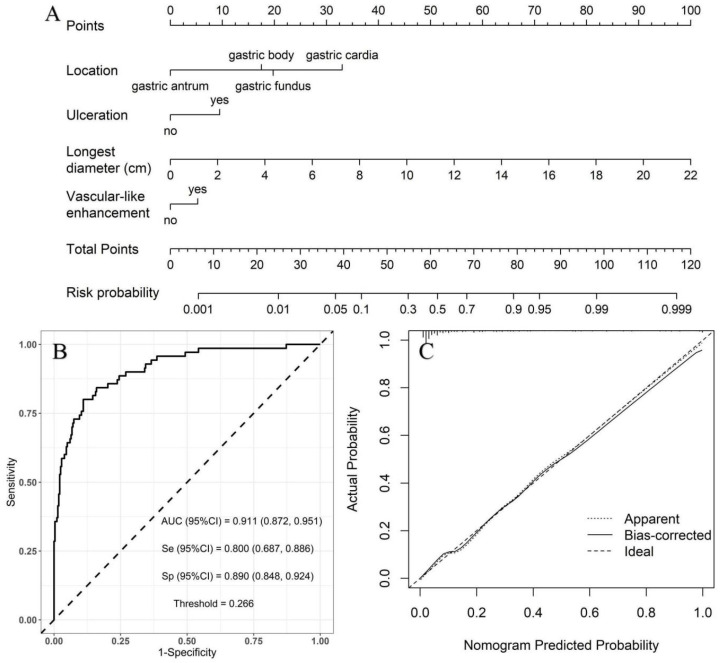
Using low-risk group as a reference, construction of predicting high-risk GSTs nomogram model. (**A**) Construction of nomogram based on the binary logistic regression analysis. The risk score was calculated using a nomogram. The optimal cut-off value of the total score was 54. (**B**) ROC curve of the prediction model; the sensitivity and specificity suggested that the nomogram model had good predictive performance. (**C**) Calibration curve of nomogram; the closer the bias-corrected line was to the ideal line, the more predictive accuracy of the nomogram model was (Brier value = 0.083).

Low- vs. high-risk group: with the low-risk group as a reference, the independent risk factors of high-risk GSTs included location, ulceration, and the longest diameter based on multivariate logistic regression analysis (Table 4). A nomogram model was constructed to predict the high-risk GSTs using R software (Figure 3A). The AUC value of the predictive model was 0.911 (95% CI: 0.872–0.951), and the sensitivity and specificity were 80.0% and 89.0%, respectively. The AUC obtained from the internal validation using the bootstrap method was 0.913 (95% CI: 0.870–0.947), and the sensitivity and specificity were 84.3% and 84.0%, respectively. The optimal cut-off value of the total score was 54 (Figure 3B). The ROC curve and the calibration curve (Brier value = 0.083, Figure 3C) both suggested that the nomogram model had good predictive performance.

**Figure 4:**
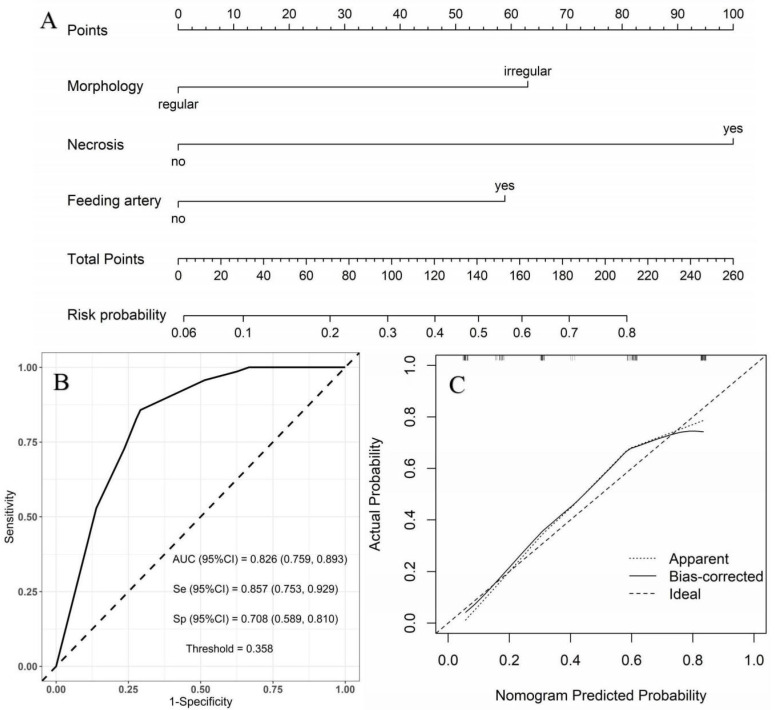
Using moderate-risk group as a reference, construction of predicting high-risk GSTs nomogram model. (**A**) Construction of nomogram based on the binary logistic regression analysis. The risk score was calculated by nomogram. The optimal cut-off value of the total score was 111. (**B**) ROC curve of the prediction model; the sensitivity and specificity suggested that the nomogram model had good predictive performance. (**C**) Calibration curve of nomogram; the closer the bias-corrected line was to the ideal line, the more predictive accuracy of the nomogram model was (Brier value = 0.163).

Moderate- vs. high-risk group: using the moderate-risk group as a reference, multivariate logistic regression analysis showed that morphology, necrosis, and feeding artery were independent risk factors of high-risk GSTs (Table 5). A nomogram model was constructed to predict the high-risk GSTs using R software (Figure 4A). The AUC value of the predictive model was 0.826 (95% CI: 0.759–0.893), and the sensitivity and specificity were 85.7% and 70.8%, respectively (Figure 4B). The AUC obtained from the internal validation using the bootstrap method was 0.828 (95% CI: 0.761–0.892), and the sensitivity and specificity were 85.7% and 70.8%, respectively. The optimal cut-off value of the total score was 111. The ROC curve and the calibration curve (Brier value = 0.163, Figure 4C) both suggested that the nomogram model had good predictive performance.

## 4. Discussion

GSTs develop from the Cajal mesenchymal cells or their common stem cells and are potentially malignant. They could occur at any age but mainly in middle-aged and seniors, exhibiting similar incidence in men and women [14], which agrees with our study.

In this study, the tumor size in the high-risk group was larger than the other two groups. Using the low-risk group as a reference, the multivariable analysis indicated that the longest diameter was the independent risk factor for high-risk GSTs. Previous studies [8,15] indicate a correlation between larger tumor size and a worse patient prognosis, which is consistent with our results. The characteristics of tumor rapid growth are indicative of malignant tumors. The tumor size in the low-risk group was similar to that of the moderate-risk group. Therefore, tumor size could not be used to differentiate low- from moderate-risk GSTs.

In the present study, we found that the incidence of ulceration increased with increasing risk classification, with significant differences between different groups. Meanwhile, ulceration was the independent risk factor for high-risk GSTs. We speculated that GSTs with a higher risk classification were probably more invasive and easily destroyed gastric mucosa. The incidence of feeding artery and vascular-like enhancement in the high-risk group was about three times higher than in the other two groups. Research by Xu et al. [16] also showed that feeding artery and vascular-like enhancement were more likely to occur in high-risk GSTs. Neovascularization is an essential step in tumor metastasis and the invasion of malignant tumors. A relatively larger tumor in the high-risk group likely accounted for this result because larger tumors would need more neovascularization to provide nutrition for tumor growth.

The necrosis rate (92.9%) of tumors in the high-risk group was significantly higher than that in the low- and moderate-risk groups. Grazzini et al. [11] also found that the necrosis rate of the tumor was 99% in the high-risk group. High-risk tumors grew faster and disproportionately to the relatively slow growth rate of neovascularization, leading to ischemic necrosis. Previous studies [16,17] reported that the degree of enhancement in the venous phase reduced as the risk stratification increased, which is in agreement with the results of the present study. A possible explanation for this outcome is that the growth rate of neovascularization in the moderate- and high-risk groups was lower than that of the tumor, resulting in relatively few contrast agents entering the tumor. In addition, the high-risk tumor is accessible to myxoid change and ischemic necrosis, which resulted in a reduced CT value in the venous phase. A previous study by Jumniensuk et al. [18] showed that GISTs with myxoid change likely exhibit recurrence and metastasis.

The growth patterns of the high-risk lesions were mainly mixed and exophytic patterns and were rarely endophytic, which is consistent with our results [8,19]. Most GSTs in the high-risk group were of irregular morphology (78.6%). On the contrary, GSTs with regular morphology were seen more often in the low- and moderate-risk groups. Neill et al. [20] also showed that irregular morphology or lobulation was an independent risk factor for GST recurrence and metastasis, which is consistent with our study. A probable explanation for this result is that tumor cell heterogeneity increases concomitantly with increasing tumor aggressiveness, contributing to a faster growth rate in more aggressive tumor parts. Fat-positive signs around the lesion were more common in the high-risk group than in the other groups. Kim et al. [21] also found that GSTs with mesenteric fat infiltration were likely highly risky. This clinical outcome might be explained by the high ability of highly aggressive tumors to infiltrate the surrounding tissue. Alternatively, a larger tumor volume in the high-risk group might compress the surrounding blood vessels more easily, resulting in adipose tissue edema.

To the best of our knowledge, there are few nomogram models of CT features for predicting risk stratification for GSTs. In the present study, using the low-risk group as a reference, multivariate logistic regression analysis showed that the location, ulceration, longest diameter, and vascular-like enhancement were independent risk factors of high-risk GSTs. With the moderate-risk group as a reference, the morphology, necrosis, and feeding artery were independent predictors of high-risk GSTs. The AUC obtained from the internal validation using the bootstrap method was basically consistent with the results of the predicted model, which indicates the stability of the model. Two nomogram models were both successfully established to predict the high-risk GSTs and had good prediction efficiency. A logistic regression equation can graphically be present with a nomogram. It is convenient and straightforward to utilize a nomogram for risk of GSTs prediction through a simple addition operation, with practical value for the clinical evaluation of patients.

There were some limitations in the study. First, a retrospective study led to a selection bias. A possible non-uniformity in the CT scanner and the parameters, injection speed, and doses of contrast agent might also have impacted the results. Second, the study failed to perform a three-dimensional reconstruction of the CT images because of a lack of thin-slice CT images, which might also have impacted the results.

## 5. Conclusions

In summary, GSTs can be classified as high risk and non-high risk with MSCT features, such as the longest diameter, tumor location, morphology, necrosis, ulceration, feeding artery, and vascular-like enhancement. The nomogram model can better distinguish the risk classification of GSTs before surgery.

## Figures and Tables

**Table 1:** AFIP criteria of GSTs.

Tumor Parameters		Characterization of Risk for Metastasis	Proportion of Patients with Progressive Disease (%)
Size	Mitotic Count		
>2 cm ≤ 5 cm	≤5 per 50 HPFs	Very low	1.9
>2 cm ≤ 5 cm	>5 per 50 HPFs	Moderate	16
>5 cm ≤ 10 cm	≤5 per 50 HPFs	Low	3.6
>5 cm ≤ 10 cm	>5 per 50 HPFs	High	55
>10 cm	≤5 per 50 HPFs	Moderate	12
>10 cm	>5 per 50 HPFs	High	86

**Table 4:** Results of binary logistic regression analysis of low- vs. high-risk group.

Risk Factor		β Value	Standard Error	Wald Value	*p* Value	OR Value (95% CI)
Location	Gastric antrum *					
Gastric fundus		2.476	1.146	4.671	0.031	11.895 (1.259, 112.345)
Gastric cardia		4.135	1.572	6.914	0.009	62.467 (2.865, 1361.919)
Gastric body		2.191	1.135	3.728	0.054	8.946 (0.967, 82.716)
Ulceration	No *					
	Yes	1.190	0.384	9.586	0.002	3.286 (1.547, 6.980)
Longest diameter	No *					
	Yes	0.569	0.090	40.251	<0.001	1.767 (1.482, 2.106)
Vascular-like enhancement	No *					
	Yes	0.658	0.419	2.468	0.116	1.931 (0.850, 4.390)

Note: * represents refer.

**Table 5:** Results of binary logistic regression analysis of moderate- vs. high-risk group.

Risk Factor		β Value	Standard Error	Wald Value	*p* Value	OR Value (95% CI)
Morphology	Regular *					
	Irregular	1.256	0.436	8.288	0.004	3.511 (1.493, 8.255)
Necrosis	No *					
	Yes	1.994	0.554	12.938	<0.001	7.342 (2.478, 1.757)
Feeding artery	No *					
	Yes	1.173	0.433	7.357	0.007	3.233 (1.385, 7.549)

Note: * represents refer.

## Data Availability

All the data were collected from the Institutional Picture Archiving and Communication System (The Affiliated TCM Hospital of Southwest Medical University and Zhongshan Hospital, Fudan University).

## Abbreviations

AFIP	Armed Forces Institute of Pathology
NIH	National Institutes of Health
GSTs	Gastric stromal tumors
GISTs	Gastrointestinal stromal tumors
MSCT	Multi-slice CT
AUC	Area under the receiver operating characteristic curve
TKI	Tyrosine kinase inhibitor
ΔCT_arterial_	CT value increment in arterial phase
ΔCT_venous_	CT value increment in venous phase
